# From sleep apnea to arrhythmia: p-wave parameters as non-invasive predictors

**DOI:** 10.3389/fcvm.2025.1623688

**Published:** 2025-09-09

**Authors:** Wenjing Lu, Ke He, Jun Zhang, Ying Deng, Shaopeng Lin, Demei Yang, Zhuojun Chen, Xinzhong Li, Xiaobo Huang

**Affiliations:** ^1^ Department of Cardiology, Nanfang Hospital, Southern Medical University, Guangzhou, China; ^2^ Department of Cardiology, The Fifth Affiliated Hospital of Sun Yat-sen University, Zhuhai, China; ^3^ Department of Hospital-Acquired Infection Control, The Fifth Affiliated Hospital of Sun Yat-sen University, Zhuhai, China; ^4^ Department of Otorthinolarynglogy, The Fifth Affiliated Hospital of Sun Yat-sen University, Zhuhai, China; ^5^ Guangdong Provincial Key Laboratory of Cardiac Function and Microcirculation, Guangzhou, China

**Keywords:** p-wave parameters, p-wave duration, Macruz Index, ptfV1, obstructive sleep apnea-hypopnea syndrome, complex atrial arrhythmias

## Abstract

**Background:**

Numerous studies have confirmed a significant association between obstructive sleep apnea hypopnea syndrome (OSAHS) and both the increased prevalence and incidence of atrial fibrillation (AF). This study advanced the endpoint event to complex atrial arrhythmias, a group that potentially develops into AF. It innovatively used non-invasive P-wave parameters (PWPs) as predictive indicators for the occurrence of complex atrial arrhythmias in OSAHS, thereby achieving early identification.

**Methods:**

A retrospective analysis was performed on the medical records of patients presenting with sleep disorders who were admitted to the Fifth Affiliated Hospital of Sun Yat-sen University between June 2019 and June 2022. Based on their apnea-hypopnea index (AHI), patients were categorized into control, mild, moderate, and severe OSAHS groups. Clinical characteristics, PWPs, occurrences of atrial arrhythmias, and echocardiographic findings were collected for analysis. Using the Kleiger grading method, atrial arrhythmias were classified into simple and complex groups. Analysis of variance (ANOVA) was employed to examine differences among the groups, while independent sample *t*-tests were used for pairwise comparisons. Logistic regression analysis was applied to identify independent risk factors contributing to complex atrial arrhythmias, and receiver operating characteristic (ROC) curves were generated to evaluate the predictive value of PWPs.

**Results:**

Patients with severe OSAHS exhibited significantly prolonged P-wave duration (PWD) and elevated Macruz Index compared to those with mild or moderate OSAHS (*p* < 0.01), while the P terminal force in lead V1 (PtfV1) was notably higher in moderate and severe groups relative to the mild and control groups (*p* < 0.01). Logistic regression analysis identified PtfV1 (odds ratio [OR] = 1.61) and the Macruz Index (OR = 2.95) as independent predictors of complex atrial arrhythmias. Furthermore, ROC curve analysis demonstrated that both PtfV1 and the Macruz Index had moderate predictive value, with area under the curve (AUC) values of 0.701 and 0.681, respectively, for identifying complex atrial arrhythmias.

**Conclusion:**

PWPs, especially the PtfV1 and Macruz index, provide a powerful non-invasive predictor of atrial arrhythmia risk in patients with OSAHS.

## 1 Introduction

OSAHS is characterized by recurrent episodes of breathing cessation during sleep, each lasting more than 10 s and occurring over 30 times within a 7-h sleep period, or by an apnea-hypopnea index (AHI) of five or more events per hour (1, 2). This disorder, primarily resulting from the collapse of the upper airway, triggers physiological changes such as elevated heart rate, increased blood pressure, and negative intrathoracic pressure, ultimately leading to hypoxemia, hypercapnia, and disrupted sleep architecture (3). These pathophysiological effects manifest in excessive daytime sleepiness, heightened cardiovascular risk, and progressive damage to multiple organs, thereby decreasing both life expectancy and overall quality of life (4). Globally, OSAHS is a prevalent condition, particularly among individuals who are overweight, obese, or elderly, with an estimated prevalence ranging from 3% to 7% in men and 2% to 5% in women (5).

Recent studies have established a strong association between OSAHS and cardiac arrhythmias, particularly AF. Patients with OSAHS experience cyclical fluctuations in heart rhythm and elevated catecholamine levels, which significantly heighten the risk of tachyarrhythmias and heart failure (6). Evidence indicates that adults with OSAHS are two to four times more likely to develop AF, with approximately 39% of individuals diagnosed with atrial fibrillation (AF also exhibiting OSAHS (5). Proposed mechanisms underlying this relationship include increased negative intrathoracic pressure, as well as enhanced vagal and sympathetic nervous system activity, both of which may contribute to atrial structural and electrical remodeling, thereby amplifying the risk of AF (7, 8).

We hypothesized that simple indicators in patients with sleep disorders could predict serious arrhythmias for early identification. The 12-lead electrocardiogram (ECG) stands out as a widely utilized, cost-effective, and patient-friendly diagnostic tool (9). The P-wave on the ECG, which reflects atrial depolarization, provides valuable insights into atrial health across a range of pathological conditions. Specifically, PWPs are instrumental in identifying atrial enlargement and conduction abnormalities. A comprehensive review has affirmed that alterations in PWPs are significantly associated with adverse clinical outcomes, including atrial fibrillation, ischemic stroke, sudden cardiac death, heart failure, and cognitive impairment, underscoring their critical relevance in clinical practice (10).

In this study, we are examining a range of PWPs that reflect atrial structural and electrical activity, including P-wave duration (PWD) and P-wave area (PWA) (10). To further assess atrial electromechanical dysfunction, we analyzed two well-established electrocardiographic markers. The Macruz Index (11), calculated as the ratio of PWD to the PR segment, serves as an indicator of interatrial conduction heterogeneity and is known to increase significantly in the presence of structural atrial remodeling. Another marker, P-terminal force in lead V1 (PtfV1) (12), quantified as the product of negative P-wave duration and voltage (ms mV), reflects left atrial enlargement and delayed conduction, with abnormal values (typically ≥0.04 mm s) predicting AF risk. Notably, patients with persistent atrial fibrillation or atrial flutter were excluded from this study due to the absence of a sinus P wave, which is essential for accurate P-wave analysis. Additionally, we applied the Kleiger grading system in an innovative manner by redefining the outcome endpoint as the occurrence of complex atrial, arrhythmias, a category previously identified as having a higher likelihood of progressing to AF (13, 14).

## 2 Materials and methods

### 2.1 Study participants and general data collection

This retrospective study reviewed the medical records of 187 patients who sought treatment for sleep disorders at the Fifth Affiliated Hospital of Sun Yat-sen University between June 2019 and June 2022. All patients underwent polysomnography using the Philips Alice 6 system in accordance with the guidelines established by the American Academy of Sleep Medicine (AASM). The results were analyzed using G3 software and subsequently verified by certified physicians at the hospital's sleep center. The study was approved by the Ethics Committee of the Fifth Affiliated Hospital of Sun Yat-sen University (Approval No.: [2023] K09-1). As a retrospective analysis, the study was exempted from the requirement of obtaining written informed consent from the participants, in accordance with institutional policy.

Inclusion criteria:

1. All patients underwent a complete 7-h standard respiratory sleep monitoring, 24-h dynamic ECG monitoring, 12-lead ECG, and echocardiography within six months before or after hospitalization, with comprehensive and complete data available from all four examinations;
2. Initial diagnosis of sleep disorders in patients with no history of prior treatment;
3. Patients exhibiting a sinus rhythm on a standard 12-lead ECG;
4. Age ≥18 years.

Exclusion criteria:

1. Patients with central and/or mixed sleep apnea;
2. Use of medications or beverages that might affect sleep monitoring on the day of the test;
3. Patients with other sleep disorders;
4. Structural or functional respiratory abnormalities affecting breathing;
5. Cardiovascular structural heart disease;
6. Acute infectious diseases, severe liver or kidney dysfunction, severe electrolyte disturbances, or endocrine disorders;
7. Patients with chronic heavy alcohol use;
8. Patients with mental or psychological disorders;
9. Patients who had already undergone antiarrhythmic therapy.

The severity of OSAHS was classified into four groups based on the apnea-hypopnea index (AHI): control group (AHI < 5), mild OSAHS (5 ≤ AHI < 15), moderate OSAHS (15 ≤ AHI < 30), and severe OSAHS (AHI ≥ 30) (15).

### 2.2 Baseline data

Baseline clinical data for all patients were obtained through a retrospective review of hospital records, including variables such as age, gender, body mass index (BMI), left atrial diameter (LAD), height, weight, relevant medical history (including hypertension, diabetes, coronary heart disease, and hyperlipidemia), as well as polysomnography parameters such as the AHI, minimum oxygen saturation (SpO_2_), and average SpO_2_.

### 2.3 Analysis variables

Standard 12-lead ECGs were recorded using the MedEx MECG-300 multi-channel ECG analysis system, with a paper speed of 25 mm/s and a standard voltage of 10 mm/mV. Examinations were performed on the first day of hospitalization while patients were in a calm and relaxed state. Only ECGs exhibiting a sinus rhythm were included for analysis. PWPs were assessed using the MedEx MECG-200 12-lead simultaneous ECG analysis system, with wave forms magnified and measured precisely using an electronic caliper. The following parameters were evaluated:

1. **PWD**: Maximum P-wave duration, measured in lead II. Three P-waves were measured, and the average value was calculated (16).
2. **PWA**: P-wave area, calculated as ½ × PWD  × P-wave voltage (average of three P-wave voltage measurements in lead II (10, 17).
3. **Macruz Index**:The ratio of P-wave duration to PR segment (time between end of electric atrial systole and onset of electric ventricular systole) (11).
4. **PtfV1**: The area of the negative portion of the P-wave in lead V1 (18).

The researchers responsible for measuring the ECG variables were blinded to both the clinical information and the polysomnography data to eliminate potential bias.

### 2.4 Outcome variables

Atrial arrhythmias were assessed through 24-h dynamic ECG monitoring, during which the presence, morphology, and frequency of atrial premature beats, couplets, bigeminy, atrial tachycardia, and paroxysmal atrial fibrillation/flutter were recorded. The data were analyzed using the Biomedical Holter system (Biomedical Instruments Co. Ltd) and interpreted by experienced physicians. Atrial arrhythmias were subsequently classified according to the Kleiger grading system (13):

• **Grade 0**: No atrial premature beats.
• **Grade 1**: Occasional atrial premature beats (≤10 beats/h).
• **Grade 2**: Frequent atrial premature beats (>10 beats/h).
• **Grade 3**: Multifocal atrial premature beats.
• **Grade 4**: Paired atrial premature beats.
• **Grade 5**: Paroxysmal atrial tachycardia, atrial fibrillation, or atrial flutter.
• **Grade 6**: Multifocal atrial tachycardia.

Grades 0–2 were defined as simple atrial arrhythmias (Group I), representing relatively benign and low-risk rhythm disturbances, whereas grades 3–6 were classified as complex atrial arrhythmias (Group II), characterized by more intricate forms, higher frequencies, and an increased potential for clinical complications (19).

### 2.5 Statistical analysis

Statistical analyses and data entry were performed using SPSS version 27.0. Continuous variables with normal distribution were expressed as mean ± standard deviation (M ± SD) and compared using independent *t*-tests or analysis of variance (ANOVA), while categorical variables were presented as percentages and analyzed using chi-square (*χ*^2^) tests. Variables demonstrating statistically significant differences between groups were selected as predictors for multivariable logistic regression analysis, which included gender, BMI, AHI, LAD, SpO_2_, PWD, PtfV_1_, and the Macruz Index. The specific PWPs, PtfV_1_ and Macruz Index, were visualized using box plots to examine their distributions, followed by an evaluation of their predictive value for complex atrial arrhythmias through receiver operating characteristic (ROC) curve analysis, quantified by the area under the curve (AUC). Based on the optimal cut-off values derived from the ROC analysis, positive predictive value (PPV) and negative predictive value (NPV) were calculated. A *p*-value of less than 0.05 was considered statistically significant.

## 3 Results

### 3.1 Comparison of general data across different degrees of OSAHS

A total of 187 patients were enrolled in the study, with a mean age of 54.19 ± 12.23 years, and males comprising 74.33% (139/187) of the cohort. Significant differences were observed among patients with varying severities of OSAHS in terms of BMI, history of hypertension, male gender proportion, minimum SpO_2_, and average SpO_2_. *Post-hoc* comparisons confirmed that these differences were statistically significant across the groups, as presented in Table 1. AHI severity was significantly associated with PWD, PtfV1, and Macruz Index at the 0.01 level. Patients with severe OSAHS exhibited significantly higher PWD and Macruz Index values compared to those in the control, mild, and moderate groups, while patients with moderate and severe OSAHS demonstrated significantly elevated PtfV_1_ values relative to the control and mild OSAHS groups.

**Table 1:** Comparison of general data Among patients with different degrees of OSAHS.

Characteristic	Control (*n* = 38)	Mild (*n* = 50)	Moderate (*n* = 42)	Severe (*n* = 57)	*χ*^2^/F	*P*	*Post-hoc* comparison
Age	54.79 ± 12.62	54.86 ± 11.43	50.93 ± 13.08	55.63 ± 11.87	1.349	0.26	
Gender							
Male	24 (63.16)	31 (62.00)	34 (80.95)	50 (87.72)	12.791	0.005	III > C; II > I; III > I
Female	14 (36.84)	19 (38.00)	8 (19.05)	7 (12.28)			
BMI (kg/m^2^)	26.02 ± 5.36	26.56 ± 3.99	27.50 ± 3.76	29.61 ± 4.93	6.119	0.001	III > C; III > I; III > II
Hypertension (*n*, %)	12 (31.58)	23 (46.00)	18 (42.86)	42 (73.68)	19.081	0.000	III > C; III > I; III > II
PWD (ms)	108.68 ± 10.02	110.68 ± 11.53	112.43 ± 12.18	122.46 ± 15.52	11.855	0.000	III > C; III > I; III > II
PtfV1 (ms*mv)	1.44 ± 1.10	1.51 ± 1.10	2.34 ± 2.40	2.47 ± 2.06	4.334	0.006	II > C; III > C; II > I; III > I
PWA (ms*mv）	5.11 ± 2.54	4.74 ± 2.14	5.39 ± 2.01	5.02 ± 2.17	0.66	0.578	III > C; III > I; III > II
Macruz Index	1.80 ± 0.62	1.96 ± 0.68	2.05 ± 0.65	2.49 ± 1.27	5.553	0.001	III > C; III > I; III > II
LAD (mm)	32.74 ± 4.00	34.26 ± 3.06	35.52 ± 3.22	36.67 ± 4.16	9.767	0.000	II > C;III > I;III > C;
Minimum SP0_2_	84.68 ± 8.18	82.58 ± 6.85	78.17 ± 8.51	74.75 ± 9.40	13.712	0.000	C > II; C > III; I > II > III
Average SP0_2_	94.00 ± 1.73	93.63 ± 1.82	93.34 ± 1.29	92.50 ± 2.96	4.41	0.005	C > III; I > III

Mild: I Moderate: II Severe: III Control: C.

### 3.2 Comparison of general data among different atrial arrhythmia groups

As presented in Table 2, out of the 187 patients included in the study, 98 individuals (52.41%) developed complex atrial arrhythmias (Group II), with grade 4 arrhythmias being the most frequently observed. In contrast, among patients with simple atrial arrhythmias (Group I), 80.90% (72 out of 89) exhibited grade 1 arrhythmias. Comparative analysis revealed that Group II had significantly higher proportions of male patients, elevated BMI, AHI, and LAD, along with lower minimum SpO_2_ and higher values for PWD, PtfV_1_, and the Macruz Index compared to Group I (Table 3).

**Table 2:** Frequency distribution of atrial arrhythmias according to kleiger classification.

Atrial Arrhythmia Group	Kleiger Grade	Frequency (n)	Percentage (%)
Group I (*n*=89)	0	14	7.49
	1	72	38.50
	2	3	1.60
Group II (*n*=98)	3	22	11.76
	4	37	19.79
	5	28	14.97
	6	11	5.88
**Total**		187	100

**Table 3:** Comparison of general data between different atrial arrhythmia groups.

Characteristic	Group I (*n*=89)	Group II (*n*=98)	*χ*^2^/*t*	*p*
Male, *n* (%)	58 (65.17)	81 (82.65)	7.473	0.006
BMI (kg/m^2^)	26.76 ± 4.67	28.34 ± 4.68	−2.301	0.022
AHI	11.70 ± 13.56	33.61 ± 22.38	−8.179	0.000
PWD (ms)	110.02 ± 10.75	118.10 ± 15.19	−4.227	0.000
PtfV1 (ms*mV)	1.37 ± 1.15	2.53 ± 2.13	−4.675	0.000
Macruz Index	1.82 ± 0.57	2.37 ± 1.09	−4.438	0.000
LAD (mm)	33.67 ± 3.52	36.14 ± 3.89	−4.538	0.000
Minimum SpO_2_	81.22 ± 9.36	78.18 ± 8.73	2.299	0.023

### 3.3 Analysis of predictive variables for complex atrial arrhythmias

Multivariablelogistic regression analysis was conducted to identify predictors of complex atrial arrhythmias, with significant differences between groups in variables such as gender, BMI, AHI, LAD, minimum SpO2, PWD, PtfV1, and the Macruz Index. The occurrence of complex atrial arrhythmias was the dependent variable. The results showed that AHI (OR = 1.07, 95% CI: 1.04–1.10, *p* < 0.01), PtfV1 (OR = 1.61, 95% CI: 1.17–2.21, *p* < 0.01), Macruz Index (OR = 2.95, 95% CI: 1.54–5.67, *p* < 0.01), and LAD (OR = 1.15 95% CI: 1.02–1.29, *p* < 0.05) were all significant risk factors for complex atrial arrhythmias (Table 4). There was no colinearity among the variables of model (Variance inflation factors, VIF < 2, tolerence > 0.2).

**Table 4:** Multivariable logistic regression analysis for predictors of Complex atrial arrhythmiasy.

Predictive Factor	*β*	SE	Wald *χ*^2^	*p*	OR (95% CI)
Constant	−11.116	3.892	8.158	0.004	0 (0.00–0.048)
Gender (Female vs. Male)	−0.247	0.457	0.292	0.589	0.78 (0.32 −1.91)
BMI	−0.023	0.050	0.207	0.649	0.98 (0.89–1.08)
AHI	0.066	0.016	18.046	0.000	1.07 (1.04 −1.10)
PWD (ms)	−0.009	0.018	0.241	0.624	0.99 (0.96 −1.03)
PtfV1 (ms*mV)	0.476	0.161	8.703	0.003	1.601 (1.17 −2.21)
Macruz Index	1.082	0.333	10.555	0.001	2.95 (1.54 −5.67)
LAD (mm)	0.136	0.058	5.475	0.019	1.15 (1.02 −1.29)
Minimum SpO_2_ (%)	0.047	0.029	2.548	0.110	1.05 (0.989–1.11)

### 3.4 Predictive value of PtfV1 and Macruz index for complex atrial arrhythmias

**Figure 1:**
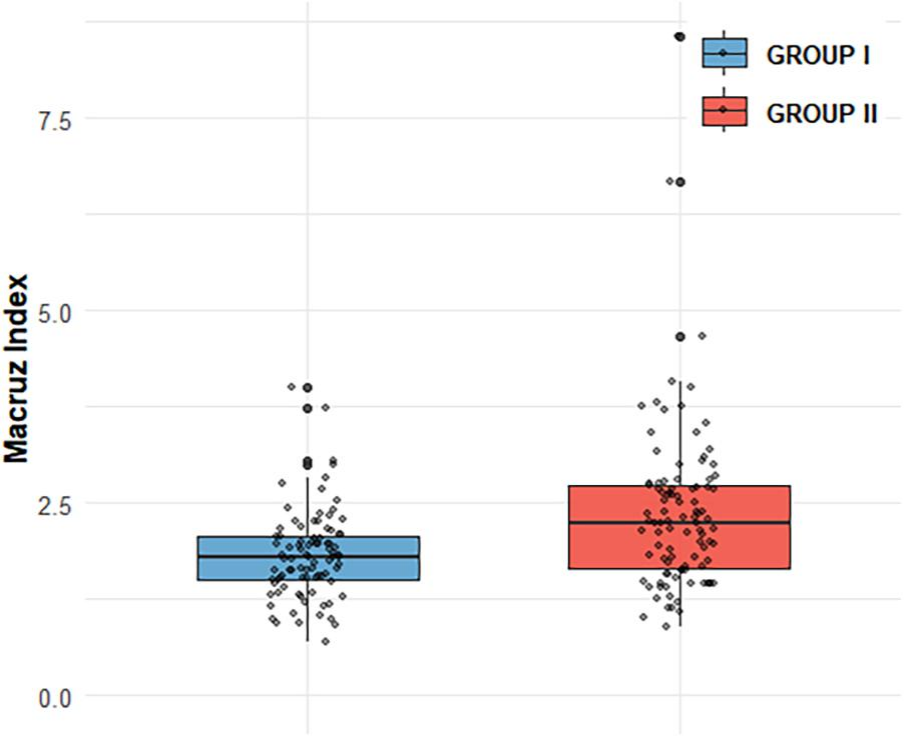
Comparison of Macruz Index distribution between GROUP I and GROUP II Box plot shows Macruz Index distribution: GROUP I (*n* = 89, mean = 1.82, median = 1.79, IQR = 0.57); GROUP II (*n* = 98, mean = 2.38, median = 2.24, IQR = 1.07). Scatter points are individual data. Box edges mark IQR, inner line is median, box length reflects IQR.GROUP II has higher mean/median (2.38/2.24 vs. 1.82/1.79) and larger IQR (1.07 vs. 0.57), indicating greater central tendency and variability.

**Figure 2:**
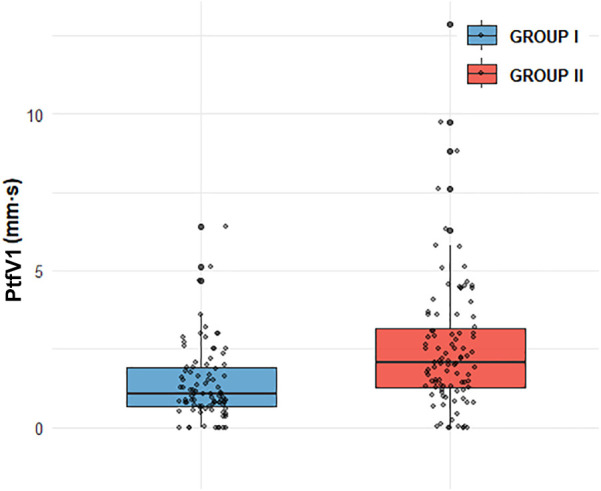
Comparison of PtfV1 distribution between GROUP I and GROUP II GROUP I (*n* = 89, mean = 1.37, median = 1.05, and IQR = 1.89),GROUP II (*n* = 98, mean = 2.52,median = 2.055, IQR = 3.17). GROUP II exhibits a higher mean (2.52 vs. 1.37) and median (2.055 vs. 1.05), alongside a larger IQR (3.17 vs. 1.89), indicating both greater central tendency and data variability compared to GROUP I.

**Figure 3:**
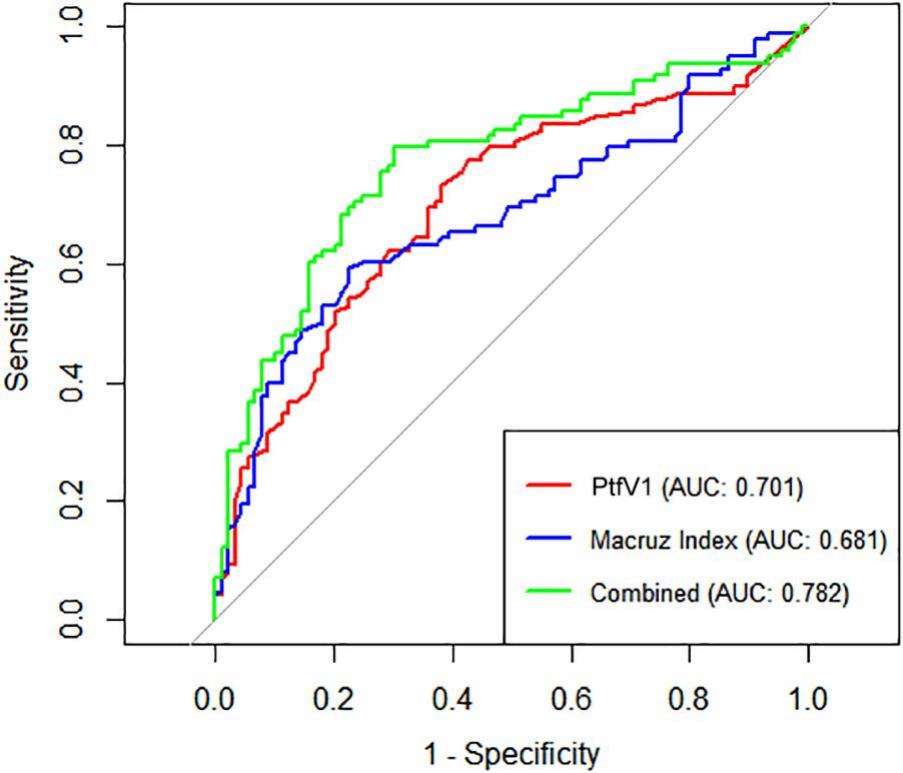
ROC curves for predicting Complex atrial arrhythmias ROC curves show diagnostic performance of PtfV1, Macruz Index, and their combination for complex atrial arrhythmias. *X*-axis: 1-Specificity (false-positive rate); *Y*-axis: Sensitivity (true-positive detection of arrhythmias). Curves: PtfV1 (red, AUC = 0.701), Macruz Index (blue, AUC = 0.681), combined (green, AUC = 0.782). Higher AUC (closer to 1) indicates better efficacy. The combined index outperforms singles, with the best balance of true/false positives. Diagonal (AUC = 0.5) is random reference; greater deviation means stronger value.

Box plot analysis revealed significantly higher values of PtfV_1_ and the Macruz Index in patients with complex atrial arrhythmias (Group II) compared to those with simple arrhythmias (Group I), with both differences reaching statistical significance (*p* < 0.001; Figures 1, 2). The predictive value of PtfV_1_ and the Macruz Index for identifying complex atrial arrhythmias was further evaluated using ROC curve analysis. The AUC for PtfV_1_ was 0.701 (95% CI: 0.625–0.776, *p* < 0.01), and for the Macruz Index, it was 0.681 (95% CI: 0.604–0.758, *p* < 0.01). When both parameters were combined, the AUC increased to 0.782 (95% CI: 0.713–0.850, *p* < 0.01), indicating enhanced predictive power. The optimal cutoff value for PtfV_1_ was determined to be 1.26 ms mV, yielding a sensitivity of 74.5% and specificity of 80.9%. For the Macruz Index, the optimal threshold was 2.077, with a sensitivity of 59.2% and specificity of 77.5% (Figure 3). Based on these thresholds, PtfV_1_ demonstrated a positive predictive value (PPV) of 80.2% and a negative predictive value (NPV) of 84.5% for identifying patients at high risk of complex atrial arrhythmias, while the Macruz Index showed a PPV of 75.6% and an NPV of 78.9% (Table 5).

**Table 5:** The positive predictive value (PPV) and negative predictive value (NPV) of PtfV1 and Macruz Index.

ECG Indicator	PPV	NPV
PtfV1	66.1	68.4
Macruz Index	74.4	63.3

## 4 Discussion

This study aimed to identify non-invasive predictors of complex atrial arrhythmias in patients with OSAHS using standard electrocardiographic tools. A total of 187 patients presenting with difficulty falling asleep were analyzed, with comparisons made between those in the control group (AHI < 5) and those with varying degrees of OSAHS severity. Our findings underscore the clinical utility of PWPs, particularly PtfV_1_ and the Macruz Index, as effective indicators for identifying patients at elevated risk of developing complex atrial arrhythmias. The four data points collected (ECG, PSG, echocardiogram, and Holter monitoring) are non-invasive, affordable, and feasible. Additionally, we accounted for variables affecting the OSAHS severity and atrial arrhythmias, boosting the reliability of the results.

According to the American Heart Association, OSAHS arises from the interaction between upper airway anatomical characteristics and sleep-related changes in airway function, and is closely linked to cardiovascular conditions such as hypertension and heart failure (20). The significant health and economic burden posed by OSAHS and its associated comorbidities highlights the urgent need for early risk stratification. ECG changes can serve as physiological markers of respiratory events, aiding not only in the detection of OSAHS but also in assessing its severity (21). Recent advances have demonstrated the effectiveness of deep learning models using single-lead ECG signals to classify OSAHS severity with high accuracy (22). In alignment with these technological developments, our study utilizes the more widely available 12-lead ECG to investigate electrophysiological markers predictive of arrhythmic risk in patients with OSAHS.

Apnea-induced hypoxemia and hypercapnia activate the sympathetic nervous system, leading to elevated heart rate and peripheral vasoconstriction (23). This recurrent autonomic stress, coupled with intrathoracic pressure fluctuations during apneic episodes, contributes to myocardial remodeling and dysfunction (24). These pathophysiological changes specifically increase left atrial (LA) afterload, promote structural alterations such as fibrosis, and prolong atrial conduction time, reflected electrophysiologically through changes in P-wave alterations (25). The resulting increase in sympathetic tone and atrial structural remodeling associated with OSAHS facilitates the onset of atrial arrhythmias, particularly AF. Our findings are consistent with this mechanism: patients with OSAHS who developed complex atrial arrhythmias exhibited significantly prolonged PWD and enlarged left atrial diameter (LAD). Multivariable logistic regression analysis confirmed both LAD (OR = 1.15, *p* < 0.05) and PWD as significant risk factors, supporting the hypothesis that OSAHS-induced atrial stretch and conduction delay contribute to arrhythmogenesis. This is further corroborated by follow-up observations in which several patients with severe OSAHS progressed to AF. Notably, continuous positive airway pressure (CPAP) therapy has been shown to reduce AF recurrence in this patient population (26).

An international consensus document endorsed by the International Society for Holter and Noninvasive Electrocardiology (ISHNE) has acknowledged the predictive value of PWPs in certain cardiovascular conditions (10), suggesting a future direction in which alterations in P-wave characteristics may serve not only as reflections of atrial structural and electrical activity but also as indicators of underlying pathophysiological changes during disease progression. Our findings reinforce this potential within the context of OSAHS-associated arrhythmias. To ensure measurement accuracy, we utilized advanced ECG analysis software capable of automatic PWP calculation, which enhanced waveform magnification for precise boundary detection. Given the limitations and variability associated with manual P-wave measurements, the use of such software significantly improved reliability by clearly identifying the onset and offset of the P-wave and PR segment (27). In the domain of cardiac electrophysiology, excitation initiated by the sinoatrial node produces the P-wave, with its first half representing right atrial depolarization and the latter half corresponding to left atrial depolarization. Anatomical changes such as right atrial enlargement can increase P-wave amplitude, while left atrial enlargement often results in prolonged PWD. Ultimately, the morphology of the P-wave during sinus rhythm is shaped by complex interactions between anatomical structures, electrophysiological properties, and geometric orientations (28).

**Figure 4:**
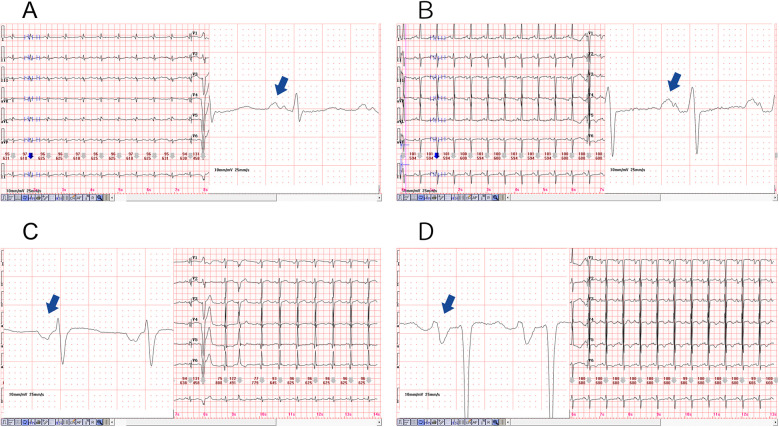
Diagram of prolonged P-wave duration in the two representative cases, magnification of the lead II revealed a significantly prolonged PWD, as illustrated in **(A,B)**. The PWD is greater than 120 (ms), and a biphasic pattern was also evident. Furthermore, magnification of the lead V1, as depicted in **(C,D)**, resulted in the observation of a deep and wide negative wave.

PWD serves as a direct and reliable measure of atrial conduction time, with numerous studies demonstrating a positive correlation between both the presence and severity of OSAHS and P-wave prolongation observed on a 12-lead ECG. This prolongation, coupled with uneven sinus node excitation, may predispose patients to atrial arrhythmias (29). Apneic episodes impose increased mechanical and pressure load on the left atrium, leading to structural remodeling and prolonged PWD, and have been identified as independent predictors of atrial scar formation (30). Histopathological investigations further link P-wave morphology changes to conduction disturbances and fibrofatty infiltration of atrial tissue. Specifically, delayed conduction through Bachmann's bundle, referred to as interatrial block, can result in atrial remodeling and asynchronous atrial contraction (31). In our study, 98 out of 187 patients developed complex atrial arrhythmias, with this group demonstrating a significantly higher incidence of PWD prolongation compared to those with simple arrhythmias. The observed prolongation reflects a delay in atrial conduction likely associated with hypoxia-induced fibrotic changes, supporting prior research findings. Moreover, structural remodeling of atrial tissue and electrical conduction abnormalities appear to be key contributors to the complexity and persistence of these arrhythmias (Figure 4).

**Figure 5:**
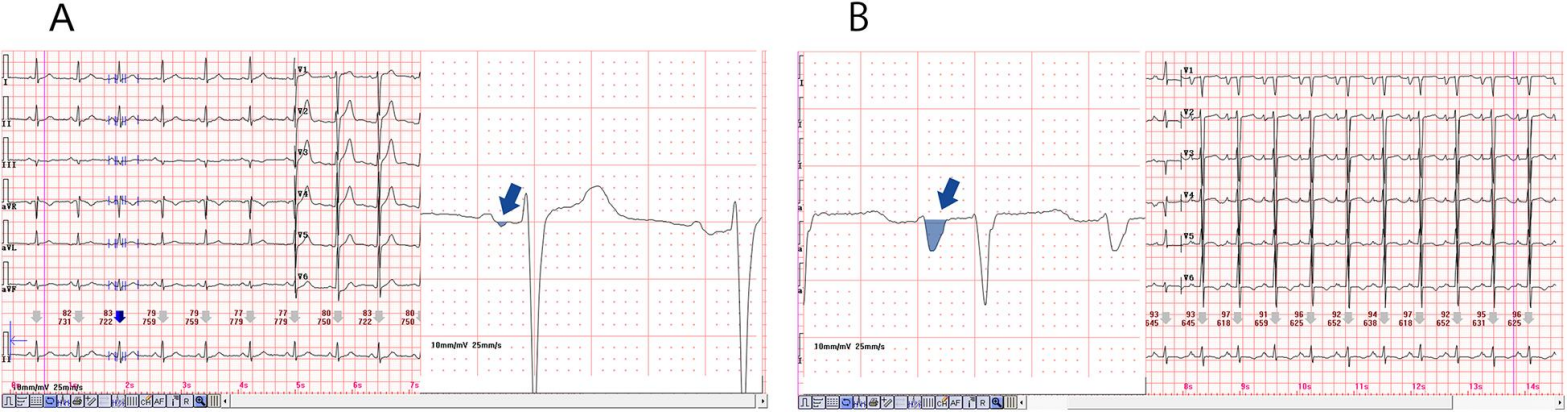
Diagram of the normal and abnormal PtfV1. The P-wave in lead V1 of a 12 lead electrocardiogram is usually positive and negative in both directions. P-wave terminal force in lead V1 refers to the product of the horizontal axis (ms) and vertical axis (mV) of the negative part. **(A)** is the Ptfv1 of a normal patient, and **(B)** is a significantly increased PtfV1, usually greater than 4 (ms mV).

PtfV_1_ demonstrates a moderate correlation with left atrial size and has been associated with a range of cardiovascular conditions, including sudden cardiac death. It represents left atrial activation via Bachmann's bundle, typically producing a negative deflection in lead V_1_ on the ECG (24, 32). Increasingly, researchers are investigating PtfV_1_ to explore the pathophysiological link between OSAHS and AF. Elevated PtfV_1_ values in OSAHS patients are indicative of abnormal left atrial electrophysiological activity, which may explain the association between OSAHS and adverse cardiovascular outcomes such as AF and stroke (17). Importantly, PtfV_1_ has also been shown to predict ischemic stroke independently of AF, highlighting the clinical significance of left atrial abnormalities in thromboembolic risk (33). AF, one of the most common arrhythmias in adults, is closely associated with increased risks of stroke, heart failure, cardiovascular mortality, and sudden cardiac death (34). In our study, patients with moderate and severe OSAHS exhibited significantly higher PtfV_1_ levels than those in the control and mild OSAHS groups (Figure 5). Furthermore, logistic regression analysis confirmed PtfV_1_ as an independent and significant predictor of complex atrial arrhythmias. (OR = 1.61, 95% CI: 1.17–2.21, *p* < 0.01), reinforcing its diagnostic and prognostic value in identifying OSAHS patients at increased arrhythmic risk.

In this study, we investigated the novel application of the Macruz Index, defined as the ratio of PWD to the PR segment, in patients with OSAHS. First introduced by Macruz and Perloff in 1958, this ECG-derived index was originally proposed as a simple method for detecting atrial enlargement (35). The Macruz Index has since attracted growing clinical interest for its diagnostic value. In cases of right atrial enlargement, the PWD typically remains stable, while the PR segment increases, resulting in a lower Macruz Index. Conversely, left atrial enlargement is characterized by prolonged PWD relative to the PR segment, which leads to an elevated index value, often exceeding the normal upper limit of 1.6 (11). This physiological basis underscores the index's sensitivity to left atrial conduction disturbances. Previous research has demonstrated significantly higher Macruz Index values in patients with multiple sclerosis compared to healthy controls, further indicating its utility in detecting subclinical atrial abnormalities. Moreover, a marked reduction in the Macruz Index has been reported following successful percutaneous mitral balloon valvuloplasty, suggesting its responsiveness to structural and functional cardiac changes (36). Recent studies have also established a strong association between elevated Macruz Index values and AF recurrence following radiofrequency ablation, highlighting its potential as a cost-effective and practical predictor for post-ablation AF recurrence (37).

In our study, compared with Group I, patients in Group II exhibited higher levels of several clinical indicators, including BMI, AHI, LAD, PWD, PtfV1, and the Macruz index, particularly among male patients, while the minimum SpO2 value was notably lower. These disparities suggest that chronic intermittent hypoxia and fluctuating intrathoracic pressure in OSAHS patients contribute to left atrial dilation and fibrosis, pathological changes that prolong PWD, elevate PtfV1, and amplify the Macruz index. An elevated PtfV1 typically reflects increased left atrial pressure, whereas a heightened Macruz index is more indicative of prolonged atrial conduction time. Enlargement of the left atrial anterior-posterior diameter offers a structural substrate conducive to the development of atrial arrhythmias, underscoring the mechanisms of atrial remodeling in OSAHS. The box plot analysis further illustrates that median PtfV1 in Group II was significantly elevated (median = 2.055 ms mV, IQR = 3.17) with greater variability compared to Group I (median = 1.05 ms mV, IQR = 1.89). Similarly, the Macruz index in Group II demonstrated higher mean and median values (2.38/2.24 vs. 1.82/1.79) and a broader interquartile range (1.07 vs. 0.57), indicating increased central tendency and dispersion. ROC curve analysis revealed that both PtfV1 and the Macruz index possess clinical utility in predicting complex arrhythmias in OSAHS patients: PtfV1 showed the highest specificity (80.9%), while the Macruz index, despite a lower AUC value (0.681), maintained good specificity (77.5%) and moderate sensitivity (59.2%). While PtfV1 demonstrates moderate predictive capacity, the Macruz index offers superior positive predictive value but is slightly less effective in ruling out negative cases, suggesting its greater suitability for identifying high-risk individuals, whereas PtfV1 is better suited for excluding those at lower risk. Collectively, these findings support the use of PtfV1 and the Macruz index in tandem as robust indicators for stratifying the risk of complex atrial arrhythmias in patients with OSAHS.

### 4.1 Limitations

This study has several limitations that should be acknowledged. The relatively small sample size (*n* = 187) and retrospective design limit the statistical power and generalizability of the findings, while also introducing the potential for selection bias. Additionally, the absence of a standardized protocol with predefined timepoints for conducting key assessments, such as ECG, PSG, echocardiography, and Holter monitoring, impedes accurate temporal analysis of the relationships among these diagnostic tools. The observed associations between PWPs and atrial remodeling remain speculative in the absence of histological confirmation, and the exclusive reliance on ECG data without incorporating quantitative respiratory measures, such as the burden of hypoxemia, restricts the depth of pathophysiological insights specific to OSAHS.

### 4.2 Future directions

Future prospective multicenter cohort studies should adopt standardized assessment protocols that define precise timepoints for conducting ECG, PSG, echocardiography, and Holter monitoring in relation to diagnosis and treatment, in order to clarify the temporal dynamics of these parameters. Such studies should also incorporate comprehensive polysomnographic data, quantitative histological evaluation of atrial fibrosis, and detailed P-wave analysis to more accurately identify electropathological correlates specific to OSAHS (38).

## 5 Conclusion

This study demonstrates that PWPs derived from 12-lead ECG, particularly PtfV1 and the Macruz index, possess significant predictive value for identifying patients with OSAHS who are at elevated risk for developing complex atrial arrhythmias. While the study has certain limitations, the findings lay a valuable foundation for enhancing risk stratification and guiding early clinical interventions in this high-risk population.

## Data Availability

The original contributions presented in the study are included in the article/Supplementary Material, further inquiries can be directed to the corresponding authors.
